# Surgical reconstruction of the left main coronary artery with patch-angioplasty

**DOI:** 10.1186/1749-8090-6-24

**Published:** 2011-03-04

**Authors:** Ivo Martinovic, Hans Greve

**Affiliations:** 1 Department of Cardiothoracic Surgery, Philipps University Marburg, Germany; 2 Department of Cardiothoracic Surgery - Klinikum Krefeld - Germany

## Abstract

**Background:**

Conventional coronary artery bypass grafting (CABG) has been established as the treatment of choice for left main coronary artery (LMCA) stenosis However, the conventional grafting provides a retrograde perfusion to extensive myocardial area and leads prospectively to competitive flow of the non-occluded coronaries thus consuming the grafts. Surgical reconstruction of the LMCA with patch-angioplasty is an alternative method that eliminates these drawbacks.

**Methods:**

Between February 1997 and July 2007, 37 patients with isolated LMCA stenosis were referred for surgical ostial reconstruction. In 27 patients (73%) surgical angioplasties have been performed. All patients were followed up clinically and with transesophageal echocardiography (TEE) and coronary angiography when required.

**Results:**

In 10 patients (27%) a LMCA stenosis could not be confirmed. There were no early mortality or perioperative myocardial infarctions. The postoperative course was uneventful in all patients. In 25 patients, TEE demonstrated a wide open main stem flow pattern one to six months after reconstruction of the left main coronary artery with one patch mild aneurysmal dilated.

**Conclusions:**

The surgical reconstruction with patch-angioplasty is a safe and effective method for the treatment of proximal and middle LMCA stenosis. Almost one third of the study group had no really LMCA stenosis: antegrade flow pattern remained sustained and the arterial grafts have been spared. In the cases of unclear or suspected LMCA stenosis, cardio-CT can be performed to unmask catheter-induced coronary spasm as the underlying reason for isolated LMCA stenosis.

## Introduction

It has been estimated that isolated left main coronary artery (LMCA) stenosis accounts about 1% of all cases of coronary artery disease [1,2]. Isolated ostial stenosis of the LMCA is mostly caused by atherosclerotic plaques [3]. Idiopathic fibromuscular hyperplasia and inflammatory diseases such as post-radiation aortitis, syphilitic- and Takayasu aortitis are rare causes of the LMCA stenosis [4,5]. Isolated LMCA stenosis are diagnosed usually by coronary angiography, but this investigation itself can cause main coronary artery spasms [6]. If these patients are treated by drugs, the 4- and 6-year survivel rates are 65 and 44% respectively [7]. Despite of increasing catheter-based procedures with PTCA and stenting of the LMCA, the results lead clearly to conclusion that the surgical treatment, conventionally by coronary bypass surgery remains the procedure of choice. Patch angioplasty was introduced in 1965 by Sabiston and colleagues, as well as Effler and colleagues [8,9], but was soon abandoned because of the high operative mortality. Hitchock et al revived the concept 20 years later and suggested that angioplasty is a valuable alternative to CABG with excellent results [10]. Since then, only few small groups have been enthusiastic reported with very good results. However, patch angioplasty has not been accepted as a standard method of treatment. Long-term results regarding the patency rate and clinical outcome after the patch LMCA angioplasty, contrary to CABG, are limited.

The aim of the present study was to review the mid to long-term outcomes of LMCA ostial reconstruction with saphenous vein patch at our center.

## Patients and Methods

The study group consisted of 37 patients with isolated LMCA stenosis referred for surgical ostial reconstruction, of 7200 patients who underwent surgical coronary revascularization at our center between February 1997 and July 2007. Age averaged 73 (46-87). 27 patients, (73%), were male. All patients were followed up clinically and with transesophageal echocardiography (TEE) yearly. Because of unclear chest pain in five patients, without changes in the ECG and TEE and the ischemia in one patient, six patients were followed up with coronary angiography.

### Preoperative findings

Preoperative angiography demonstrated significant ostial LMCA stenosis in all patients.

Predominant aortic valve stenosis was present in 9 patients, predominant aortic valve incompetence in 2 patients. Diffuse, non significant coronary disease was present in 6 patients. Myocardial bridge over the LAD was present in one patient. Mild mitral valve incompetence was diagnosed in 3 patients. Left ventricular ejection fraction (LVEF) was good in 15, moderate in 9 and poor in 3 patients.

## Operative Technique

**Figure 1:**
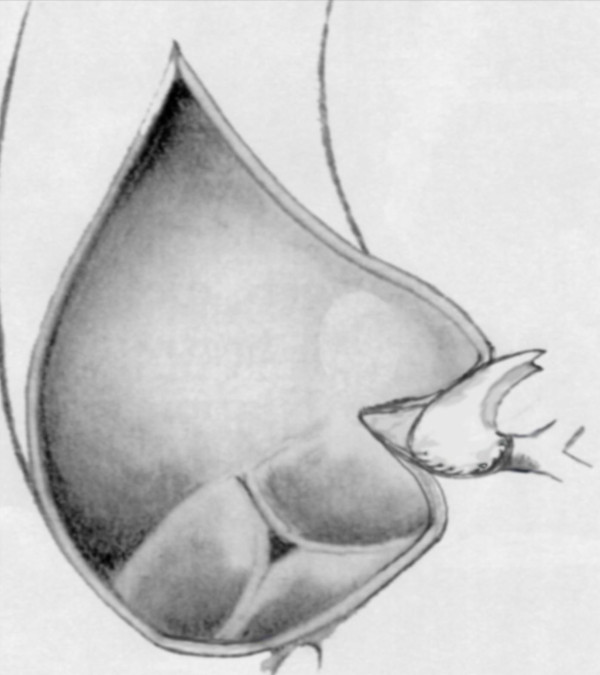
**Patch-angioplasty with autologous saphenous vein**.

Cardiopulmonary bypass, moderate hypothermia, cold blood antegrade and retrograde cardioplegia with topical cooling were used in all patients. The left ventricle was vented by a catheter advanced through the right superior pulmonary vein. We divided the pulmonary artery in four cases in order to improve the exposure. An aortic incision was used, beginning on the anterior medial wall of the aorta. Both ostia were inspected and carefully checked out with a 5 mm probe. In the patients with confirmed LMCA stenosis, the left main stem was reconstructed. The LMCA reconstruction technique has been decribed before by Ridley [11]. After the aortic cross clamping was completed, the main pulmonary trunk was encircled with a silicone loop and retracted laterally. The pericardial fat was removed from the LMCA before incising the coronary ostium. The aortic incision was directed into the ostium of the LMCA and extended through the LMCA to the bifurcation of the circumflex and left anterior descending artery branches. Ostial reconstruction was performed with a saphenous vein in all patients. The vein patch was used to enlarge not only LMCA but also the area of the aortic incision (Figure 1). A continuous 7-0 polypropylene was used to create a new funnel-shaped LMCA segment and a continuous 5-0 polypropylene suture was used onto the adjacent aortic wall.

### Operative findings

**Figure 2:**
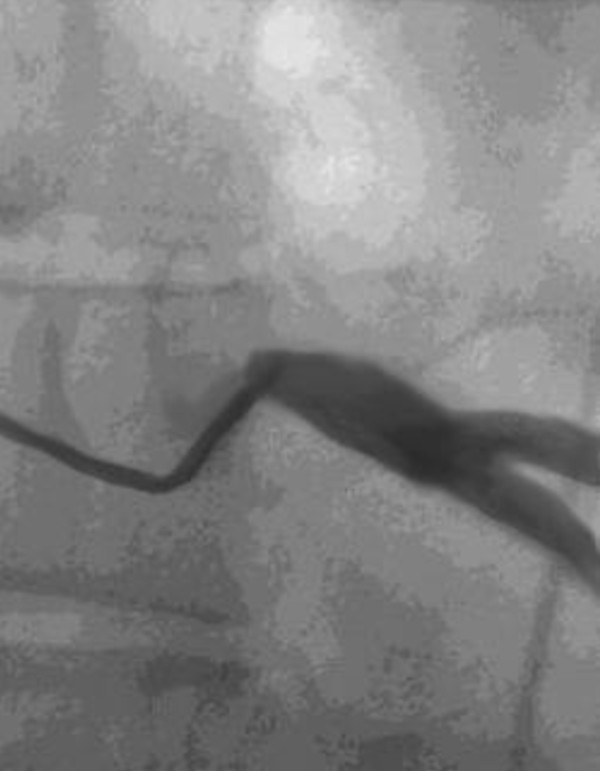
**LMCA stenosis**.

**Figure 3:**
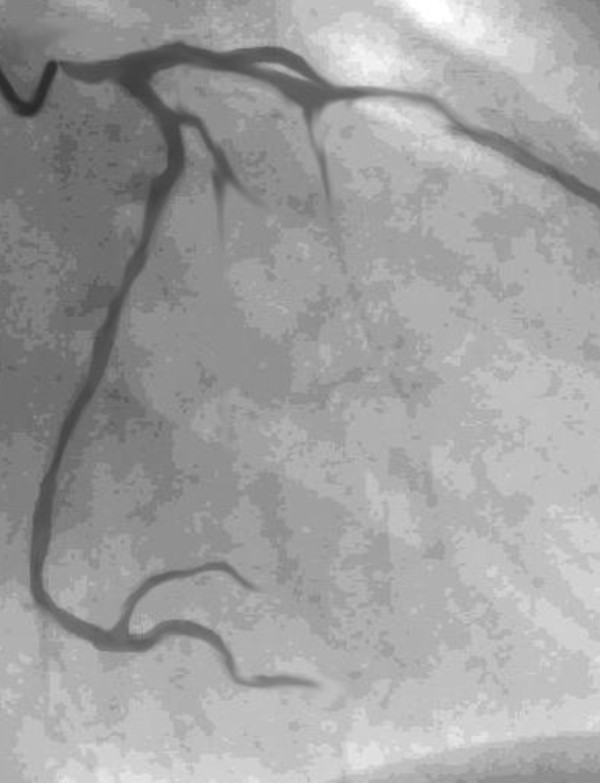
Spasm of the ostial LMCA

In 10 patients, a LMCA stenosis could not be confirmed; the LMCA ostium was easily passed by the 5 mm probe. One of them had long stenosis with a muscle bridge over the left anterior descending artery, which was released by incision of myocardium. In other 9 patients, one venous graft to LAD was performed for safety reasons. In 27 cases the ostium stenoses were confirmed and enlarged with saphenous vein patch. In two patients with massive calcification, an endarterectomy had to be performed. In one of these patients, two bypass grafts were performed in addition to the main stem angioplasty. Figure 2 shows the coronary angiography of the patient with intraoperatively confirmed LMCA stenosis. Narrowing of the left coronary ostium by spasm is seen on Figure 3. In one female patient, endarterectomy with patch enlargement was not performed, because calcification included the bifurcation, thus a conventional bypass revascularization was performed.

The average aortic cross-clamp time was 34 &(plusmn) 14 minutes (range 24-62 minutes, the CPB time 54 &(plusmn) 16 minutes (range 43-87 minutes), and the total duration of the operation 113 &(plusmn) 14 minutes (range 93-136 minutes).

## Results

### Early results

The operation was uneventfull in all but one patient, who developed signs of ischemia in the ECG after disconnecting the extracorporal circulation. The branches of the left coronary artery were grafted in addition and IABP was started.

The postoperative course was uneventful in all cases. There was no in-hospital death. No postoperative myocardial infarction was observed. The mean stay in the ICU was 11.2 hours and the mean hospital stay 6.4 days. There were no significant clinical complications.

### Follow-up

**Figure 4:**
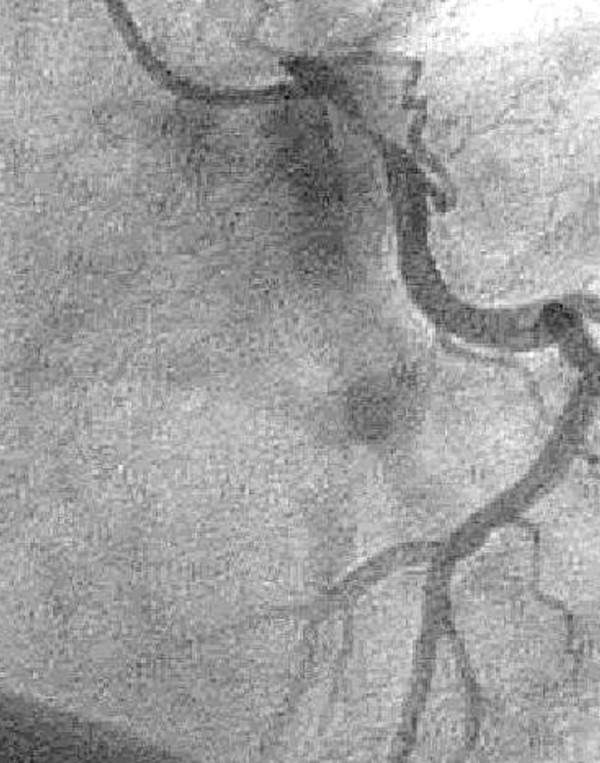
**Stenosis after patch angioplasty**.

All patients underwent follow-up clinical examination and transesophageal echocardiography (TOE) yearly. TOE demonstrated a wide open LMCA physiological flow pattern in all patients with not confirmed LMCA stenosis. The duration of follow-up ranged from 3 to 120 months (mean 59 &(plusmn) 34 months. Normal flow pattern and none calcification of the patch were demonstrated in 25 patients. In 5 patients the angiography showed a large main stem 1 to 6 months after reconstruction of the coronary arteries. In one patient, acute coronary syndrome occurred within 6 months. The angiography showed a significant stenosis of the distal main stem at the end of the patch enlargement (Figure 4). A coronary artery bypass grafting procedure had to be performed immediately.

## Discussion

Coronary angiography is the standard procedure used to identify definitive coronary anatomy. Other methods are magnetic resonance imaging (MRI) [12] and Doppler-echocardiography [13], preferably by transesophageal approach [14]. The proximal parts of the main coronary artery can be visualized very well by intravascular ultrasound (IVUS), and by electron-beam CT scanning [15]. Invasive catheterization can lead to mechanically induced spasm of coronary arteries. In the cases of unclear or suspected LMCA stenosis, CT angiography can be performed to unmask catheter-induced coronary spasm as the underlying reason for isolated left main coronary artery stenosis seen in invasive angiography. Coronary vasospasm is defined as a decrease in the caliber of the coronary arteries with evidence of ischemia, commonly known as variant or Prinzmetal's angina. This degree of ischemia may be significant enough even in patients with normal coronaries to produce myocardial damage as indicated by elevated cardiac troponin I and cardiac arrest [16,17]. Variant angina typically occurs during rest or at night. Cigarette smoking is the major risk factor for coronary vasospasm [18]. Additional factors implicated in vasospasm include hypocalcemia, [19], sotalol [20], pseudoephedrine, [21], hyperventilation [22] and cocaine use [23]. 10 of 37 patients in our cohort, reffered for surgical reconstruction of the LMCA had no really stenosis. The LMCA could have easily passed with a 5 mm probe. 8 of them had a concomitant aortic valvular disease. Dyspnea was a predominant symptom in these patients. A heavy spasm must have been occurred during angiography. For safety reasons one vein bypass was performed as suggested by Soga et al [5]. In our opinion these patients have benefited of the plan to perform the reconstruction. Otherwise a competitive flow after CABG with patent LMCA would have consumed the grafts in this cohort very soon.

While single authors report good results of unprotected left main coronary artery PTCA and stenting, the results of the German PTCA Register show different experiences; the mortality rate was more than 9% in cases of angioplasty of unprotected LMCA stenosis and even of 4,8% in angioplasty of LMCA stenosis with very good collateralization.

These results lead to the conclusion that surgical treatment is much superior to PTCA. Ostial stenoses are treated customarily by performing coronary bypasses to both great branches of the left coronary artery. However, conventional CABG method restores less physiological, retrograde perfusion of the myocardium, may also lead to occlusion of the LMCA, can result in competitive flow and consumes bypass material. Surgical reconstruction of the LMCA avoids these potential inconveniencies, and additionally allows subsequent percutaneous coronary intervention of the distal coronary tree.

**Figure 5:**
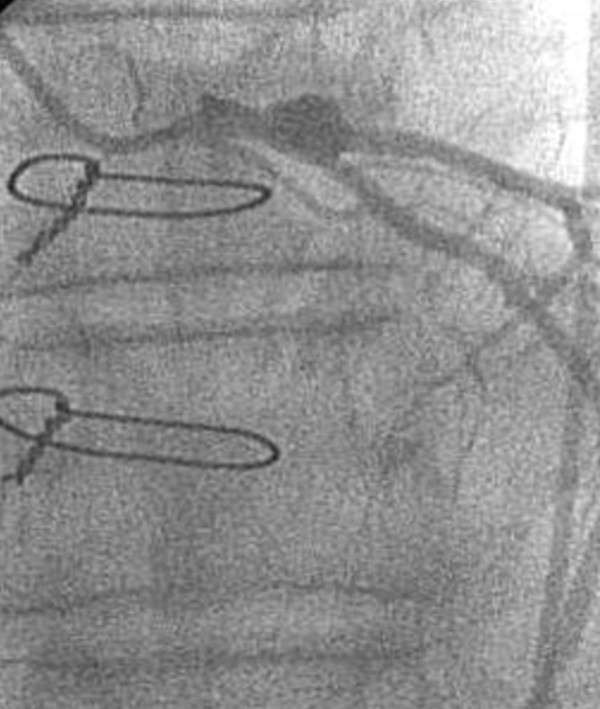
**Dilated vein patch after angioplasty**.

Several different operative approaches have been described [8,10,24-27]. In our study, we used only anterior approach as first described by Sullivan and Murphy [24]. We only used fresh autologous saphenous vein patch for reconstruction because it is simple to be sewn and is wide enough to create a funnel shaped LMCA ostium. Dion and colleagues suggested that the saphenous vein patch owing its potential fibrinolytic activity, might be preferable to autologous pericardium [28]. Theoretically, the autologous vein has a tendency to proliferative degeneration resembling that of CABG using the saphenous vein. Also, because of its elasticity, autologous vein patches tend to dilatation [29]. In our group one vein patch looked as moderate dilated and was documented in the first patient with extensive calcification in the bifurcation and endarterectomy of the distal LMCA (Figure 5). We expected this finding cause we produced it intentionally. In that patient we did not inspect the whole length of the LMCA before incising its ostium. We started incising a moderately calcified proximal LMCA and than found excessive calcification in the distal part and bifurcation. Extensive angioplasty with large vein patch was essential. However, no progression of the patch "dilatation" in the follow-up has been seen. In our series of patients there was only one patient with restenosis in the follow-up. In this patient with distal main stem stenosis, the endarterectomy was performed before reconstruction. Thus, we suggest that the presence of isolated stenosis only in the proximal and middle part of LMCA and the absence of severe calcifications should be considered as indications for surgical reconstruction. In the cases of unclear or suspected LMCA stenosis, our actual concept is to perform a cardio-CT in order to unmask catheter-induced coronary spasm as the underlying reason for isolated LMCA stenosis.

## Conclusion

The surgical reconstruction with patch-angioplasty is a safe and effective method for the treatment of the proximal and the middle LMCA stenosis. Endarterectomy and reconstruction should be avoided in the case of distal left ostial stenosis and excessive calcification. Long term follow-up is required to determine the patency in order to evaluate this method. Left ostial stenosis could not be confirmed in almost one third of the study group: antegrade flow pattern remained sustained and the arterial grafts have been spared.

## Competing interests

The authors declare that they have no competing interests.

## Authors' contributions

IM carried out the study. HG participated in the design of the study. All authors read and approved the final manuscript.
